# Central Retinal Artery Occlusion Associated with Takayasu Arteritis

**DOI:** 10.3390/diagnostics14131329

**Published:** 2024-06-23

**Authors:** Sehreen Mumtaz, Claire Wilson, Prasanna Vibhute, Eric R. Eggenberger, Florentina Berianu, Andy Abril

**Affiliations:** 1 Department of Rheumatology, Mayo Clinic Florida, Jacksonville, FL 32224, USA; berianu.florentina@mayo.edu (F.B.); abril.andy@mayo.edu (A.A.); 2 Department of Internal Medicine, Mayo Clinic Florida, Jacksonville, FL 32224, USA; wilson.claire@mayo.edu; 3 Department of Radiology, Mayo Clinic Florida, Jacksonville, FL 32224, USA; vibhute.prasanna@mayo.edu; 4 Department of Ophthalmology, Mayo Clinic Florida, Jacksonville, FL 32224, USA; eggenberger.eric@mayo.edu

**Keywords:** Takayasu arteritis, retinal artery occlusion, large-vessel vasculitis

## Abstract

Takayasu arteritis is a chronic inflammatory vasculitis with granulomatous panarteritis particularly impacting large vessels including the aorta and its branches, especially the subclavian arteries, with clinical manifestation dependent on the involved artery. Sequelae of the active disease vary, including stenosis, occlusions, or aneurysmal dilatations of the large vessels. The prevalence of Takayasu arteritis is higher in the Asian population and in Japan, but quite low in the United States, varying from 0.9–8.4 per million people. Ocular manifestations are rare and lead to a delay in diagnosis and appropriate treatment. Ocular manifestations include Takayasu retinopathy, anterior ischemic optic neuropathy (AION), retinal artery occlusion (RAO) and retinal vein occlusion (RVO). We present two cases in which central retinal artery occlusion (CRAO) was associated with Takayasu arteritis. CRAO is an ophthalmic emergency with an incidence of 1.9 per 100,000 person years in the United States; only 5% of cases are arteritic, which can be observed with inflammatory vasculitides secondary to the formation of immune deposits.

A 35-year-old woman with a prior history of left central retinal artery occlusion and bilateral sensorineural hearing loss, initially concerning with respect to neurosarcoidosis (never histologically proven), presented for clinical follow-up. She had a complicated disease course dating back many years, involving different specialists, and including fluctuating hearing loss partially responsive to corticosteroids necessitating cochlear implants and developed central retinal occlusion of the left eye (Figure 1 and Figure 2). This was concerning with respect to possible underlying inflammatory conditions such as neurosarcoidosis or Susac syndrome (MRI atypical sans corpus lesion), and she received IV immunoglobulin treatment. When she returned for follow-up, she had complaints of episodes of confusion, but quiescent symptoms without visual change, status post adalimumab and methotrexate addition. She sought emergency care for new transient arm numbness and confusion, and a neck CTA revealed carotid bulb circumferential thickening suggestive of vasculitis. A rind of soft tissue thickening involving the distal half of the common carotid artery, carotid bifurcation and the proximal internal and external carotid arteries was visualized. There was smooth narrowing of the encased carotid artery lumen, producing roughly 50% diameter stenosis of the distal common carotid artery. A small ulceration was noted on the left carotid bulb (Figure 3, Figure 4 and Figure 5). The patient was initiated on infliximab with IV methylprednisolone and close follow-up with neurology and vascular medicine for management of Takayasu arteritis.

A 56-year-old woman was diagnosed with Takayasu arteritis in 1991, and initially treated with glucocorticoids for several years. Initial symptoms included constitutional symptoms and left-sided carotidynia. She had markedly elevated inflammatory markers and anemia at disease onset, and later developed erythema nodosum. Imaging studies in the early phase of her illness revealed moderate stenosis of the right subclavian artery and marked thickening of the left common carotid. She was subsequently treated with methotrexate and cyclosporine, and eventually her disease went into remission. In 2014, an MR angiogram of the chest showed radiographic evidence of vessel wall edema in the aorta, and at that time, the patient was started on mycophenolate mofetil. In November 2017, the patient developed blurry vision and there was leakage on her fluorescein angiogram, which was felt to be nonspecific. She was taken off mycophenolate due to no evidence of active vasculitis in clinical or laboratory findings. She was in remission; however, she required ascending aorta hemiarch repair and aortic valve replacement (and was subsequently on warfarin) for a dilated aortic root of 61 mm with histopathology showing healed arteritis. Two years later, she developed a paracentral scotoma in the left eye and was diagnosed by outside Ophthalmology as having branch retinal artery occlusion by retinal imaging, with suspected embolic disease in the context of subtherapeutic anticoagulation.

Clinical characteristics of patients with ocular manifestations and Takayasu’s arteritis have been reported to be similar to those of patients without ocular manifestations with a mean age in the third decade of life and a majority of Asian origin. Ocular manifestations of Takayasu arteritis can be seen in a wide range (8.1–68%) of patients [1]. Apart from two case-based systematic reviews, the current literature is lacking in describing the extent of ocular involvement in Takayasu arteritis [1,2]. The current classification of Takayasu arteritis does not include visual changes, and consequently no ophthalmological examination is recommended, even after diagnosis, and ocular manifestations can go unnoticed. 

A study by Pasko et al. noted ocular manifestations presenting prior to diagnosis of Takayasu arteritis in 74.6% of cases [3]. This may be explained by cases in which initial manifestations included constitutional symptoms that were non-specific. Limitation of flow in the carotid artery results in chronic ischemia and hypoperfusion retinopathy, and ocular ischemic syndrome may be of consequence [4]. It remains imperative to distinguish and categorize this ocular disorder and pursue retinal angiograms.

A study from 1994 pointed to the presence of Takayasu’s retinopathy as a statistically significant poor prognostic factor [5]. Mirouse et al. associated Takayasu retinopathy independently with mortality and complication-free survival [6]. Reportedly, 64% of CRAO patients develop new vascular risk factors after a retinal occlusive event, implying this population of patients is prone to a secondary ischemic event and needs risk factor assessment [7]. Although medical management is the recommended initial management for Takayasu arteritis, patients who have vascular complications may require surgical interventions and endovascular procedures [8]. Medical management includes ocular massage, hyperbaric oxygen therapy, and intravenous tissue-type plasminogen activator; however, these are associated with risks, including mobilization of the embolus and exacerbating macular edema. Endovascular treatments, including intra-arterial thrombolysis, have shown promising results; however, they require exceptional skill to perform [9]. 

Although information on ocular ischemia surgical management for Takayasu arteritis patients is limited, case series and case reports have demonstrated favorable results of balloon angioplasty and endovascular stenting in Takayasu retinopathy patients [1]. Adopting routine fundus fluorescein angiography for surveillance in patients with Takayasu arteritis may be beneficial. Newer diagnostic techniques, especially optical coherence tomography (OCT) angiography (OCTA), and novel diagnostics to assess retina perfusion must be investigated [1].

In young patients with visual loss and deficits, it is imperative to recall Takayasu arteritis as a potential suspect, even though such cases are rare, because it can translate into a meaningful difference in outcomes with proper management. Raising ophthalmological awareness of this association and increasing education regarding pursuing comprehensive diagnostic testing is crucial. Further investigation of arteritic CRAO in Takayasu arteritis patients is needed for the development and implementation of enhanced therapeutic techniques.

## Figures and Tables

**Figure 1:**
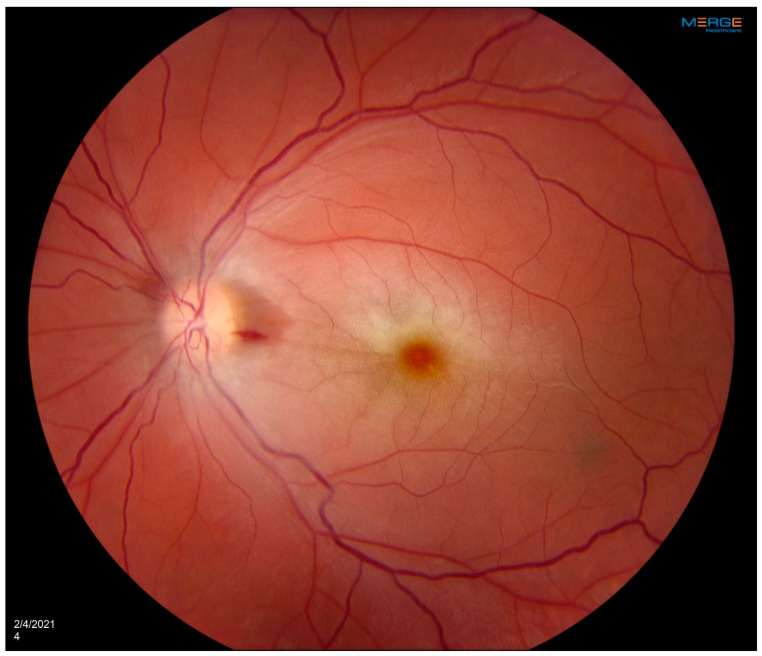
OS fundus photograph showing central retinal artery occlusion with characteristic cherry red spot and attenuated blood vessels. Patient developed left central retinal artery occlusion years prior to vasculitis diagnosis with loss of vision at a young age.

**Figure 2:**
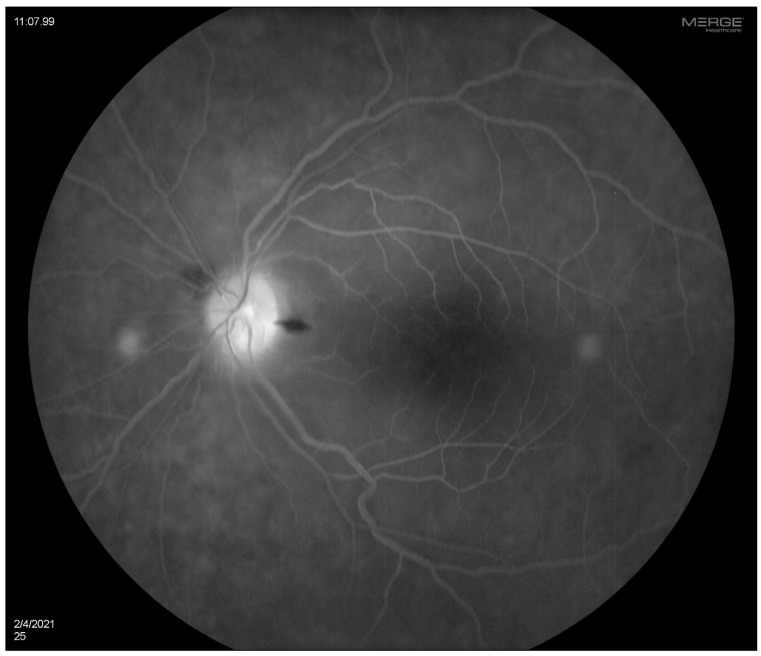
OS fluorescein angiogram showing arterial narrowing with some stain of vessels on the superior optic disc.

**Figure 3:**
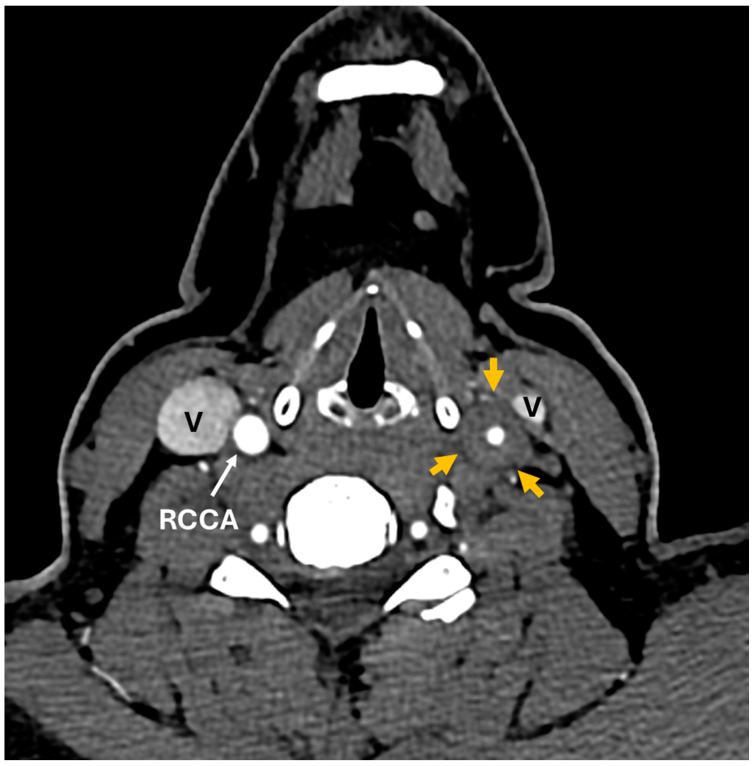
CT angiogram. Axial images below the level of carotid bifurcation show diffuse circumferential thickening of common carotid artery (CCA) and internal carotid artery (ICA) walls on the left side (orange arrows).

**Figure 4:**
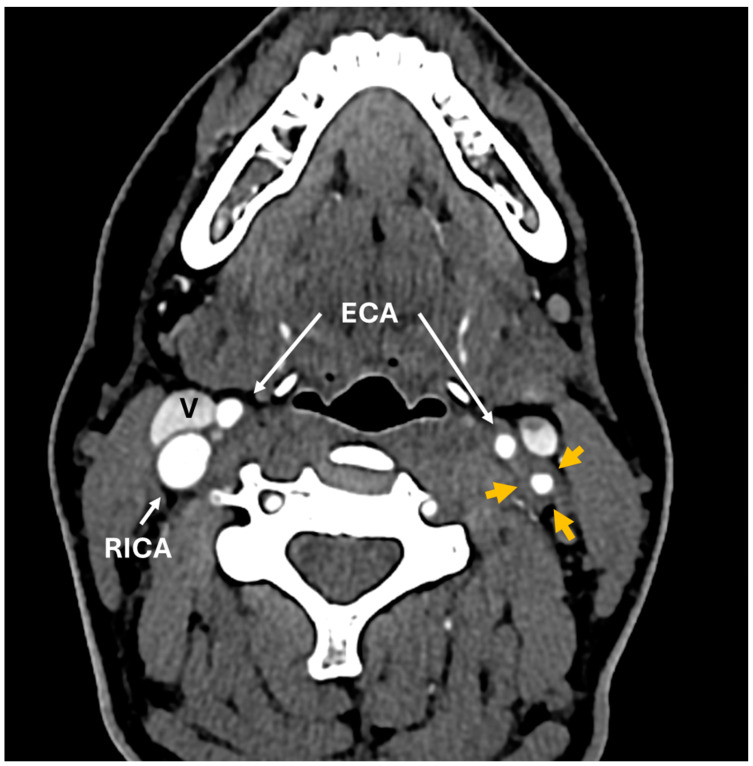
CT angiogram. Axial images above the level of carotid bifurcation show diffuse circumferential thickening of common carotid artery (CCA) and internal carotid artery (ICA) walls on the left side (orange arrows). Compare this to the normal wall thickness of the right carotid artery. White arrows point to the right common carotid artery (RCCA), right internal carotid artery (RICA) and bilateral external carotid arteries (ECAs). Internal jugular veins: (V). A repeat CT neck angiogram with contrast revealed a left-sided diffuse circumferential rim of soft tissue thickening involving the left carotid artery, which was concerning with respect to large-vessel vasculitis. It is unclear how long the carotid bulb thickening had been present.

**Figure 5:**
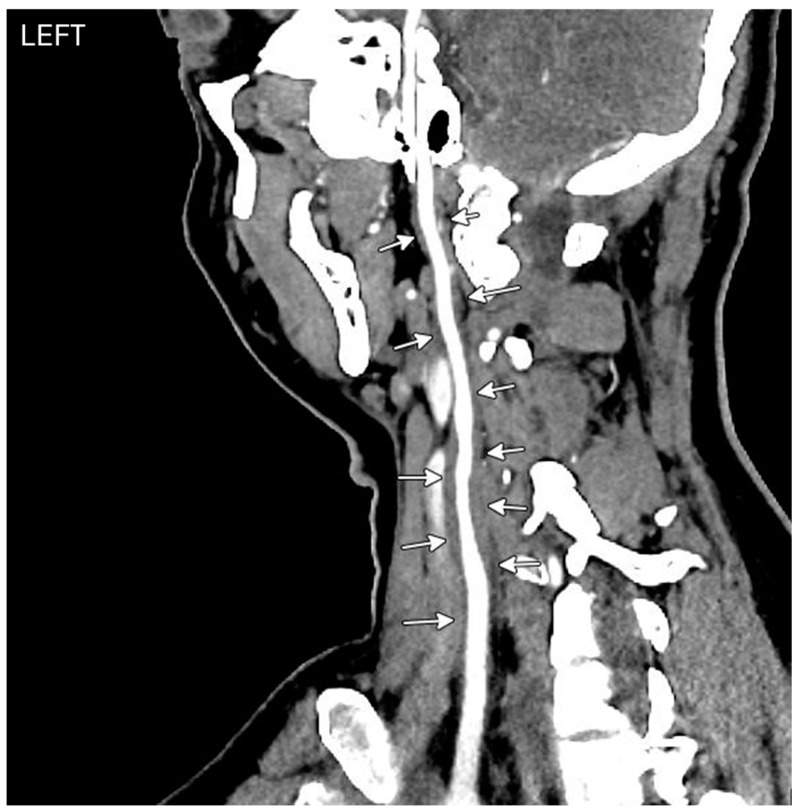
CT angiogram. Left carotid artery centerline reconstructions show diffuse arterial wall thickening (white arrows).

## Data Availability

Written informed consent has been obtained from the patient to publish this paper.
